# Spatiotopic updating facilitates perception immediately after saccades

**DOI:** 10.1038/srep34488

**Published:** 2016-09-30

**Authors:** Jasper H. Fabius, Alessio Fracasso, Stefan Van der Stigchel

**Affiliations:** 1 Experimental Psychology, Helmholtz Institute, Utrecht University, Heidelberglaan 1, 3584 CS Utrecht, The Netherlands; 2 Radiology, Center for Image Sciences, University Medical Center Utrecht, 3584 CX Utrecht, The Netherlands; 3 Spinoza Centre for Neuroimaging, University of Amsterdam, 1105 BK Amsterdam, The Netherlands

## Abstract

As the neural representation of visual information is initially coded in retinotopic coordinates, eye movements (saccades) pose a major problem for visual stability. If no visual information were maintained across saccades, retinotopic representations would have to be rebuilt after each saccade. It is currently strongly debated *what* kind of information (if any at all) is accumulated across saccades, and *when* this information becomes available after a saccade. Here, we use a motion illusion to examine the accumulation of visual information across saccades. In this illusion, an annulus with a random texture slowly rotates, and is then replaced with a second texture (motion transient). With increasing rotation durations, observers consistently perceive the transient as large rotational jumps in the direction opposite to rotation direction (backward jumps). We first show that accumulated motion information is updated spatiotopically across saccades. Then, we show that this accumulated information is readily available after a saccade, immediately biasing postsaccadic perception. The current findings suggest that presaccadic information is used to facilitate postsaccadic perception and are in support of a forward model of transsaccadic perception, aiming at anticipating the consequences of eye movements and operating within the narrow perisaccadic time window.

When inspecting the world, visual information travels from the retina to the visual cortex in a retinotopic reference frame. Yet, the eyes are continuously moving, creating large shifts in retinal images and thereby posing a serious problem for visual stability. Are perceptual representations updated across saccades, in a spatiotopic reference frame, or do they start anew upon each fixation? This question has gained increasing interest since the presentation of neurophysiological evidence of perisaccadic shifts of receptive fields, suggesting that information is exchanged between neurons around the time of saccades^1^,^2^,^3^.

As illustrated by the phenomenon of change blindness, not the entire visual scene is updated across saccades^4^. It has been argued that only the behaviourally relevant features at saccade endpoint are updated spatiotopically^5^,^6^. This presaccadic acquisition of visual features at the saccade target can then be used to predict the perceptual consequences of the eye movement at the fovea^7^,^8^,^9^, compatible with forward models where sensory processing is influenced by the predicted consequences of upcoming, self-generated movements^10^,^11^,^12^. Recently, several behavioural studies have indeed provided evidence for spatiotopic updating of feature information such as orientation, colour, shape and motion^13^,^14^,^15^,^16^,^17^,^18^.

However, others studies did not find transfer of visual features from a retinotopic into a spatiotopic representation^19^,^20^,^21^,^22^, prompting the hypothesis that *not* feature information, but only spatial information can be updated spatiotopically using ‘attentional pointers’^23^. This hypothesis has primarily been investigated using cueing effects, showing that a cue presented before a saccade is effective soon after a saccade in spatiotopic coordinates^24^,^25^,^26^. Whether this also holds for trans-saccadic integration remains unknown, as there are currently no studies addressing the time course of spatiotopic updating of perceptual representations after a saccade.

Here, we address the issue of spatiotopic visual stability by taking advantage of a recently described motion illusion – High Phi^27^ – to measure the rapid induction of a motion percept. Using this illusion, the current experiments address *whether* and *when* presaccadic visual features influences postsaccadic perception. In the High Phi illusion, an annulus with a random texture (inducer) rotates slowly clockwise or counter-clockwise, and is then replaced with several different textures (transient). With increasing inducer durations, participants report the transient more and more as large rotational jumps in the direction opposite to inducer direction (backward jumps). Importantly, the successive different textures, that trigger the illusory jump, are presented transiently, allowing for direct manipulation of perception onset. By manipulating the reference frame of the inducer with respect to the transient we were able to compare the benefit of spatiotopic representations on the speed of building a perceptual representation after a saccade. The data of two experiments are compellingly in favour of rapid spatiotopic interpretations of visual information after eye movement offset. This supports the hypothesis of a perceptual system where object representations can be updated spatiotopically across saccades, taking into account both object features and position. Thereby, this system enables fast spatiotopic interpretations of visual information immediately after saccade offset.

## Results

### Reference frame of the High Phi illusion (Experiment 1)

**Figure 1:**
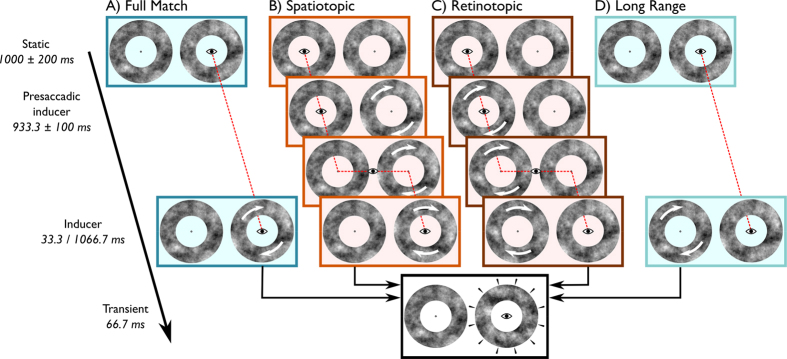
Trial sequences in Experiment 1. The small eye and red dotted line depict gaze position. White arrows on the annuli depict rotations of that particular annulus. All trials started with a period of fixation with two static annuli on screen. (**A**,**D**) In the fixation trials (Full Match and Long Range) the static annuli were followed by a rotation of one the two annuli (Inducer, 33.3 or 1066.7 ms). The annulus around fixation rotated in case of the Full Match trials, or in the periphery, in case of the Long Range trials, and was then succeeded by the presentation of series of 4 different textures (Transient). (**B)** In the Spatiotopic trials, the peripheral ring started rotating while the eyes remained fixated (Presaccadic inducer). Here, the position of the Presaccadic inducer was spatiotopically matched to the position of the Transient. After an auditory cue, a saccade was initiated to the peripheral, rotating annulus. After the saccade, the annulus rotated around fixation (Inducer), and was then replaced by the Transient. (**C**) In Retinotopic trials, the position of the Presaccadic inducer was initially around fixation, and therefore retinotopically matched to the position of the Transient. After an auditory cue, a saccade was executed and the Inducer was no longer retinotopically matched. Then, the Transient was presented centrally. In all trial types, the Transient would always be presented around the current fixation point. Subjects responded whether they perceived a clockwise or counter clockwise rotational jump, by pressing the right or the left arrow key on a standard keyboard. Note that the Inducer durations as described in this figure correspond to the inducer durations reported in Table 1 (*Methods*) and Fig. 2 that were analysed.

The fast temporal characteristics of the High Phi illusion make it an ideal tool to investigate the rapid building of a perceptual representation. However, when investigating transsaccadic perception, this is only useful when the accumulated motion information can be updated spatiotopically. Previous investigations into spatiotopic accumulation of motion information have yielded mixed results with some studies showing spatiotopic^13^,^16^ and others showing strictly retinotopic representations of motion^19^,^22^. Moreover, some of the spatiotopic effects have been accounted for by more general decision biases, irrespective of either the spatiotopic or retinotopic location of the stimulus^21^. Therefore, we examined the reference frame in which the High Phi illusion can be induced in Experiment 1. We adapted the illusion into a transsaccadic paradigm with four different trial types (Fig. 1). In our display, subjects were always presented with two annuli with different textures, thus enabling manipulation of the reference frame in which the inducer and the transient were presented.

In 12 naïve human subjects we tested the effects of an inducer on perceived jump direction when the inducer and the transient were (A) fully, (B) only spatiotopically, (C) only retinotopically or (D) not matched (we refer to the latter type as Long Range trials). Beside this spatial manipulation, we also varied the inducer duration in order to investigate the temporal development of the illusion.

In Full Match and Long Range trials (Fig. 1A,D), subjects remained fixation, enabling full control over the duration of the inducer which was set to either 0, 33.3 or 1066.7 ms (presented at a 60 Hz refresh rate). We were primarily interested in the 33.3 or 1066.7 ms conditions, but included the 0 to keep the number of trials in fixation and saccade blocks balanced (see *Methods: Data preprocessing*). In Spatiotopic and Retinotopic trials (Fig. 1B,C), inducers rotated both before and after subjects made a saccade. The duration of the postsaccadic inducer was of main interest to our analysis, as we wanted to investigate whether the presaccadic inducer affected the strength of the postsaccadic inducer. During the experiment, postsaccadic inducer duration was probed gaze-contingently with 16.7, 33.3 or 50.0 ms, randomly drawn from a uniform distribution. A posteriori, we determined the actual postsaccadic inducer duration with respect to saccade offset, as set by the native Eyelink saccade detection algorithm. In all trial types, the transient was presented immediately after the inducer, and subjects reported whether they perceived a clockwise or counter clockwise jump. These responses were coded with respect to the preceding inducer rotation direction. Forward jumps were coded as 1, backward jumps as −1. All trials that met our inclusion criteria (see *Methods: Data preprocessing*) were analysed using a linear logit mixed effects model^28^,^29^. The reported coefficients are in logits and relative to the baseline level in the model, comprising the Full Match trials with 33.3 ms of inducer.

#### Baseline High Phi effect

**Figure 2:**
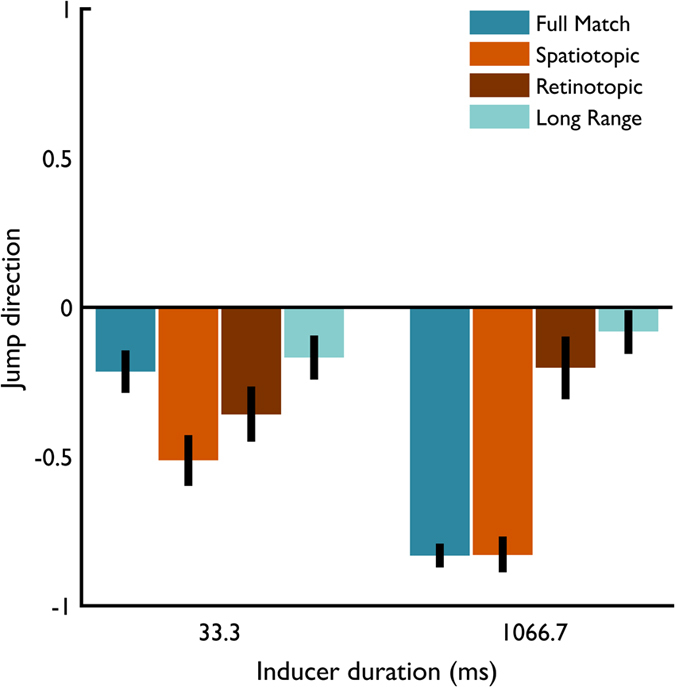
Perceived jump direction in Experiment 1 (error bars represent bootstrapped 95%-confidence intervals of the model estimates). Negative values represent backward jumps, positive values forward jumps (N = 12). In case of the Spatiotopic and Retinotopic trials, inducer duration refers to *postsaccadic* inducer duration (see Fig. 1 and Table 1). We observed a strong High Phi illusion with Full match trials (dark blue bar at 1066.7 ms of inducer duration). Moreover, the High Phi illusion could be stored retinotopically across saccades, similar to motion after effects (brown bar at 33.3 ms of inducer duration). The retinotopic effect faded out at 1066.7 ms of inducer duration, because after saccade execution the Inducer was no longer retinotopically matched with the Transient (see Fig. 1 for description of the trial sequence). Interestingly, the accumulated motion information can also be updated spatiotopically (orange bar at 33.3 ms of inducer duration). The spatiotopic effect was larger than what was observed in Full match trials (dark blue bar at 33.3 ms of inducer duration) and could not be explained as a long range or decisional bias, since the effect was larger than in the Long Range condition (light blue bar at 33.3 ms of inducer duration).

In Full Match trials, subjects fixated a single fixation point, and the positions of the inducer and the transient were fully matched, i.e. both spatio- and retinotopically. Hence, this condition, was very similar to the original paradigm of Wexler and colleagues^27^, with the addition of a second static annulus in the periphery. As depicted by the dark blue bars in Fig. 2, subjects perceived more backward than forward jumps after an inducer as brief as 33.3 ms (β = −0.44, z = 4.63, p < 0.001), and this bias grew even stronger after 1066.7 ms (β = −1.95, z = 12.54, p < 0.001). Subjects tended to perceive the changing textures (transient) as backward jumps when it was preceded by an inducer of sufficient duration.

#### Retinotopic reference frame

As was to be expected from motion aftereffect studies^19^,^22^, there was a significant bias in perceived jump direction when the inducer was only retinotopically matched to the transient that was presented 33.3 ms after saccade offset (β = −0.39, z = 2.35, p = 0.019, as compared to the Full Match 33.3 ms condition; Fig. 2, brown bars). However, this retinotopic effect wears off over time (post-hoc comparison between 33.3 and 1066.7 ms in the Retinotopic trials; β = 0.34, z = 2.16, p = 0.03), most likely because the inducer and the transient are no longer retinotopically matched after the saccade. Indeed, in a control experiment (see Supplementary Figure S1) we showed that the bias in the Retinotopic condition is as strong as in the Spatiotopic condition when the inducer motion is transferred along with the saccade (see Supplementary Figure S2).

#### Spatiotopic reference frame

Interestingly, beside a retinotopic effect, a spatiotopic effect was observed (Fig. 2, orange bars). Shortly after saccade offset, subjects reported more backward jumps than in the Full Match condition, i.e. when the postsaccadic inducer duration lasted only 33.3 ms (β = −0.69, z = 4.76, p < 0.001). This bias, like the Full Match condition grew stronger over time (post hoc comparison between 33.3 and 1066.7 ms in the Spatiotopic trials; β = 1.24, z = 5.25, p < 0.001), but the difference between a short and long inducer was not as large as in the Full Match trials (β = 0.71, z = 2.51, p = 0.012). In other words, even though the inducer rotated for 933.3 ms (on average) in the periphery before saccade onset, in Spatiotopic trials the average perceived jump direction after 33.3 ms was not as consistently backward as in the Full Match after 1066.7 ms.

#### Long range induction of High Phi

To control for potential long range effects in the High Phi illusion, or a decisional bias of a peripheral inducer on the perceived jump direction of a centrally presented transient, we examined the effect of a peripheral inducer per se, that is, without a saccade towards it. In the Long Range condition after 33.3 ms, a bias for backward jumps was observed, not significantly different from the bias observed after 33.3 ms of inducer in the Full Match condition (β = 0.10, z = 0.901, p = 0.368; Fig. 2, light blue bars). In contrast to the Full Match condition, this bias did not change with longer inducer duration (post hoc comparison between 33.3 and 1066.7 ms in the Long Range trials; β = −0.18, z = 1.68, p = 0.096). We re-ran the mixed effects model with the Spatiotopic trials as a reference level to test whether the observed long range effect is statistically different from the observed Spatiotopic effect after 33.3 ms of inducer. This model confirms that the long range effect after 33.3 ms is smaller than in the Spatiotopic condition after 33.3 ms (post-hoc β = 0.79, z = 5.45, p < 0.001). The absence of a strong long range effect is indicative for a spatially selective effect of the inducer on the perceived jump direction. The transient and the inducer have to be matched in at least a retinotopic or – and critically – a spatiotopic reference frame in order to effectively induce perceived backward jumps. Together, the results from the Long Range and Spatiotopic condition suggest that a peripherally presented inducer can effectively induce the High Phi illusion but only when the inducer becomes spatially aligned with the transient.

### Time course of spatiotopic facilitation (Experiment 2)

In Experiment 1, we showed that the High Phi illusion can be stored retinotopically across saccades, similar to motion after effects. In addition, the accumulated motion information can also be updated spatiotopically. The spatiotopic effect is present shortly after saccade offset. In Experiment 2, we addressed whether this observed spatiotopic effect is related to a faster development of the illusion, with respect to the Full Match trials (i.e. an increased effect of inducer duration in Spatiotopic trials) or to a general tendency to observe the transient as backward jumps immediately from the beginning of fixation (i.e. a change in offset in Spatiotopic trials).

The spatiotopic effect in Experiment 1 seemed to be different from a long range or decisional bias, since the observed spatiotopic effect was larger than the long range effect. However, in the Spatiotopic condition, after saccade offset, the postsaccadic inducer and the transient position were fully matched, whereas in the Long Range condition the inducer and the transient were never matched. Hence, we tested the long range effect more conservatively in Experiment 2 (see *Methods, Saccade Mimic* trials), to further control for a long range explanation of the spatiotopic effect^21^.

**Figure 3:**
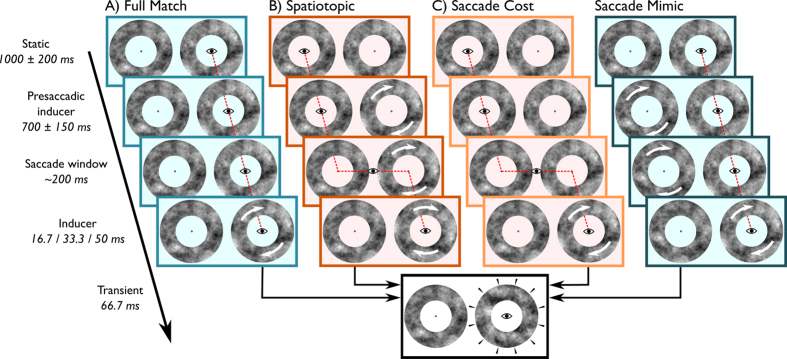
Trial sequences in Experiment 2. The small eye and red dotted line depict gaze position. White arrows on the annuli depict rotations of that particular annulus. Like in Experiment 1, all trials started with a period of fixation with two static annuli on screen. (**A**) In Full Match trials, subjects remained at fixation with the static annuli for as long as the presaccadic inducer time in Spatiotopic and Saccade Cost trials. After this period of fixation, the Inducer rotated for 16.7, 33.3 or 50.0 ms. In addition, in Full Match trials, the inducer could also rotate for 800 ms. Then, the Transient was presented. (**B**) Spatiotopic trials were similar to the Spatiotopic trials in Experiment 1. (**C**) In Saccade Cost trials subjects remained fixation and the annuli remained static up until saccade offset. A saccade was executed after an auditory cue. Upon saccade landing, the annulus around fixation started rotating (Inducer), succeed by the Transient. (**D**) In Saccade Mimic trials, the retinal input of Spatiotopic trials was mimicked, without the execution of a saccade. The Presaccadic inducer phase consisted of a peripheral rotating annulus and a central static annulus. Then, the peripheral annulus stopped rotating and the central annulus rotated for 16.7, 33.3 or 50.0 ms. In all trial types, the Transient would always be presented around the current fixation point.

12 different naïve subjects were tested on four different trial types (Fig. 3), with a higher temporal resolution with respect to inducer duration. Similar to Experiment 1, there were Full Match and Spatiotopic trials (Fig. 3A,B). Inducer duration was set to 16.7, 33.3 or 50 ms (presented at a 60 Hz refresh rate). In Full Match trials, the inducer could also rotate for 800 ms, in order to obtain a measure of the potency of the High Phi illusion at its strongest. In addition to the Full Match and Spatiotopic trials, we included Saccade Cost trials to investigate the potential cost of a saccade preceding the transient on the proportion of reported backward jumps (Fig. 3C). In these trials, subjects fixated within a static annulus, and made a saccade towards the peripheral annulus, that had also remained static. The inducer started rotating only after the saccade had ended. Hence, the retinal input was essentially similar to the Full Match trials, with the exception that a saccade was made before inducer presentation. The fourth trial type, Saccade Mimic controlled conservatively for long range effects (Fig. 3D). In these trials, a peripheral inducer rotated for 700 ms (±150, uniformly distributed), resembling the peripheral inducer duration in Spatiotopic trials, followed by an additional 200 ms (±100, uniformly distributed), approximately simulating the saccadic latency and saccadic duration in Spatiotopic trials. Then, the peripheral inducer stopped rotating and the central inducer rotated for 16.7, 33.3 or 50 ms, followed by the transient. As in Experiment 1, we analysed the perceived jump direction as a function of trial type and inducer duration with a linear logit mixed effects model.

#### Temporal development of High Phi (after saccades)

**Figure 4:**
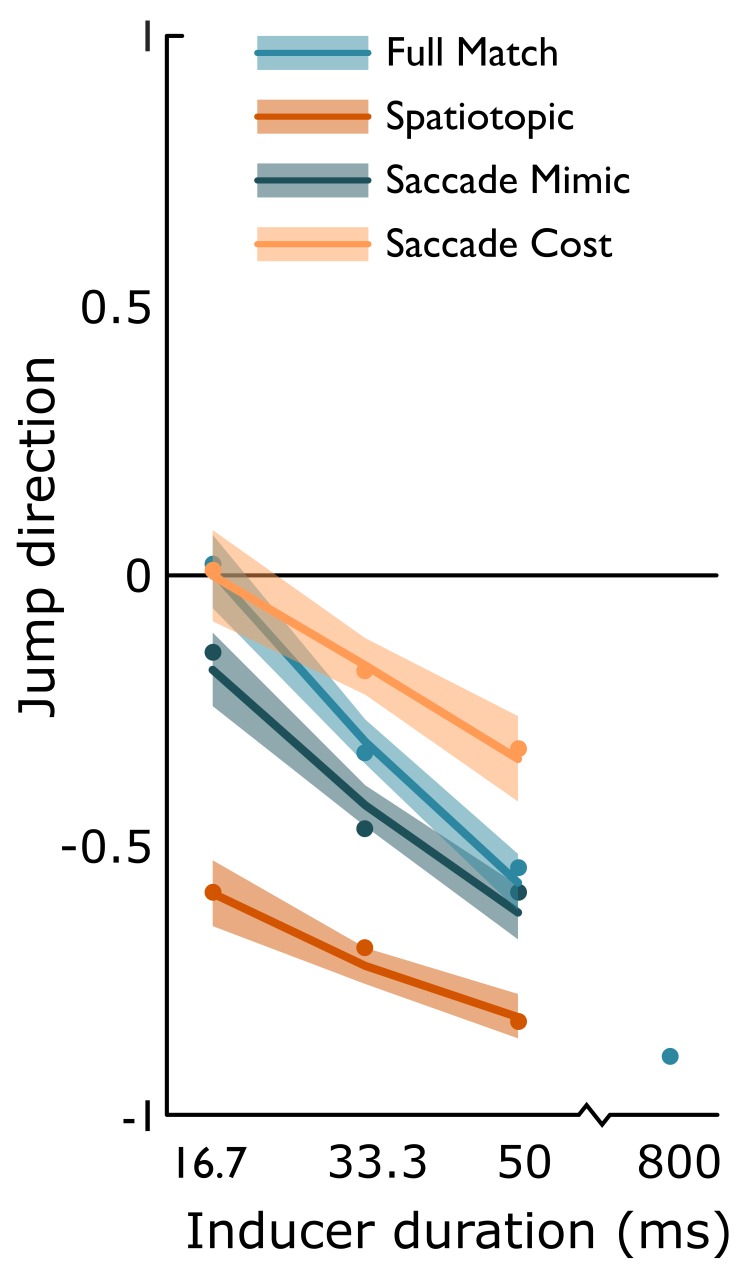
Perceived jump directions in Experiment 2. Positive values represent forward jumps, negative values backward jumps. Lines depict linear interpolation between the estimates of the linear logit mixed effects model. Shaded region represents the bootstrapped 95%-confidence intervals of the model estimates. Bootstrapped averages are depicted by the small dots (N = 12). We observed the rapid induction of the illusion, as illustrated by the slope in the **Full Match** condition (light blue), similar to the Saccade mimic condition (dark blue). In the **Saccade Cost** condition, the illusion developed more slowly over time (light orange). In the **Spatiotopic** condition, the illusion was induced more strongly than in the Full Match condition, even when the transient was presented after only a single frame of the post-saccadic inducer (dark orange line). Similarly, in the Spatiotopic condition, the illusion was induced more strongly than in the **Saccade Mimic** condition (dark blue line), even when the transient was presented after only 16.7 ms of the post-saccadic inducer.

The baseline for the development of the High Phi illusion is the effect of 16.7 ms of inducer in the Full Match condition. The rapid induction of the illusion is illustrated by the observed slope in the Full Match condition, along inducer duration (β = −0.65, z = 11.18, p < 0.001; Fig. 4, light blue line). However, in the same condition, 16.7 ms of inducer was not sufficient to induce a bias for backward jumps (β = 0.02, z = 0.15, p = 0.882). After 800 ms of inducer in the Full Match condition, the average response was −0.89 (±0.03 s.e.m.) very similar to what was observed in Experiment 1 after 1066.7 of inducer in the Full Match condition (−0.83 ± 0.04 s.e.m.). After a saccade this initial bias was similarly absent (difference between Full Match and Saccade Cost at 16.7 ms of inducer: β = 0.02, z = 0.15, p = 0.878; Fig. 4 yellow line). However, the illusion developed more slowly over time (effect of inducer duration in Saccade Cost, as compared to the effect of inducer duration in Full Match trials: β = 0.27, z = 3.01, p = 0.003), though significantly (post-hoc β = −0.27, z = 3.01, p = 0.003).

**Figure 5:**
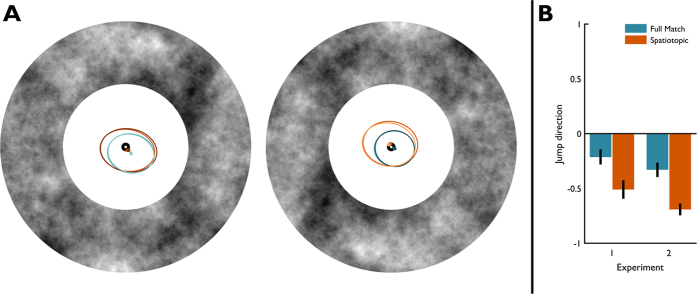
Control analyses. (**A**) Ellipses inside annuli represent the area where fixation was during transient presentation on 95% of the trials. Left and Right annuli represent Experiment 1and 2, respectively. The spread of fixation coordinates during transient presentation was larger for trials where subjects made a saccade prior to transient presentation (brown/orange/light orange ellipses). (**B**) Comparison in average perceived jump direction in Experiment 1 (N = 12) and Experiment 2 (N = 12) after 33.3 ms of (postsaccadic) inducer. The effect of a spatiotopic inducer on average perceived jump direction was stronger in Experiment 2 than in Experiment 1, yet the difference between the bias in the Full Match condition and the Spatiotopic condition was not statistically different between Experiments, even though two different samples were tested.

Note that both in the Full Match trials (Exp. 1 and Exp. 2) as well as in the Saccade Cost trials, the direction of the perceived jumps is slightly different from the results obtained by Wexler and colleagues (2013). With short inducer durations (16.7 or 33.3 ms) their subjects reported primarily forward jumps, whereas here subjects already had a slight bias to report backward jumps when a transient followed a short inducer. A potential explanation for this difference might be found in the duration of the transient (Wexler, personal communication). In their study, the transient comprised a single change in texture, here, four different textures were used. We used more textures because the perceived jump tends to increase with more different textures^27^. However, the transient duration might have also affected the direction of the perceived jump. Yet, given the internal consistency and replication of the High Phi effect in the current study (see Control Analyses and Fig. 5b), we believe the results of the Full Match condition can serve as a valid baseline for the Spatiotopic condition.

#### Temporal development after a saccade with spatiotopic preview

In contrast to the Saccade Cost condition, when a presaccadic Spatiotopic preview of the inducer was provided, the illusion was induced more strongly than in the Full Match condition (Fig. 4, orange line), even when the transient was presented after only 16.7 ms of the post-saccadic inducer (β = −1.33, z = 10.78, p < 0.001). The development of the illusion (slope) was not significantly different in the Spatiotopic condition, with respect to the slope in the Full Match condition (interaction β = 0.14, z = 1.24, p = 0.215).

#### Long range induction of High Phi

The Saccade Mimic condition was included to provide the most conservative control for the observed spatiotopic effect. In this condition a peripheral inducer rotated for at least 550 ms, and was then succeeded by a central inducer of 16.7, 33.3 or 50 ms. In these trials, there was a larger bias than in the Full Match condition after 16.7 ms inducer (β = −0.37, z = 3.74, p < 0.001; Fig. 4 dark blue line), but the slope was similar to the slope in the Full Match trials (β = 0.10, z = 1.17, p = 0.242). We re-ran the model with the Saccade Mimic trials as a reference level to further investigate the long range effects. First, this shows that the initial bias is not only stronger than in the Full Match trials, but is actually also statistically different from zero after even 16.7 ms of central inducer (post-hoc β = −0.35, z = 2.79, p = 0.005). Second, crucially, the initial bias observed after 16.7 ms of inducer in the Saccade Mimic condition was smaller than in the Spatiotopic condition (post-hoc β = 0.96, z = 7.74, p < 0.001).

### Control analyses

#### Decision bias induced by familiarity with the illusion

Beside the aforementioned long range effects, we warranted some additional caution in interpreting the Spatiotopic effect. We suspected that, given the strength of the visual illusion, subjects might have hypothesized that long inducers were always followed by backward jumps. If this were true and subjects were able to identify the inducer direction in the Spatiotopic conditions, they might have based their response on that hypothesis. To control for this effect, we ran another linear mixed effects model (for each experiment separately), where we added a random slope of Trial number within each subject. Trial number corresponded to the actual trial number that was used in the experiment. With likelihood ratio tests, we compared these new models to the original models, where Trial number was not included. Neither in Experiment 1, nor in Experiment 2 did Trial number increase the fit of the models (Exp. 1 χ^2^(2) = 4.03, p = 0.133; Exp. 2 χ^2^(2) = 4.73, p = 0.094). Therefore, we conclude that the observed Spatiotopic effect is not likely to be attributable to decision biases related to familiarity with the High Phi illusion.

#### Gaze position

In both experiments, subjects were required to make a saccade in two conditions, whereas in the other two conditions they could remain stable fixation over the entire course of the trial.

First, we analysed the average horizontal gaze positions during the transient across the different conditions. Overall, there was more variability in the conditions with saccades than in the conditions without (Fig. 5A, and see Supplementary Material). Next, we analysed the precision of fixation during transient presentation (variance in gaze coordinates during transient) and fixation error (between inducer presentation and transient presentation). When precision and error were included as random effects in our mixed effects models, inferences on the fixed effects did not change (see Supplementary Table S1). The variability in fixation precision and error does therefore not account for the observed differences in perceived jump direction across the different conditions.

#### Robustness of spatiotopic effect

Figure 5B shows the average perceived jump direction after 33.3 ms of inducer in Experiment 1 and Experiment 2. As can be clearly seen, the Spatiotopic benefit was present in both experiments, even though two different samples of subjects were used. An additional linear mixed effects analysis with Experiment and Condition as fixed effects and Subject as a random effect was ran to test this. In both samples, there was a bias in the Full Match condition after 33.3 ms of inducer motion (β = −0.45, z = 2.98, p = 0.003), and the bias was stronger in the Spatiotopic condition in both experiments, compared to the Full Match condition (β = −0.69, z = 4.63, p < 0.001). The Spatiotopic was stronger in Experiment 2 than in Experiment 1 (β = −0.64, z = −2.55, p = 0.011). However, the interaction between experimental condition (Spatiotopic and Full Match) and Experiment number was not significantly different (β = 0.37, z = 1.81, p = 0.07). Together, these results show that the trans-saccadic High Phi paradigm as developed in this study yields robust and consistent results across observers.

## Discussion

We investigated whether and when presaccadic visual information is integrated with postsaccadic information. Using the fast temporal dynamics of the High Phi illusion, we demonstrated that presaccadically acquired information influences perception immediately after a saccade. We excluded potential long range explanations of this spatiotopic facilitation with several control conditions. Our data support the hypothesis of a perceptual system that uses predictions based on presaccadic information to efficiently process postsaccadic information^8^,^12^. These predicted consequences are commonly thought to enable the cancellation of self-generated changes from external changes in visual input, as observed in saccadic suppression of intrasaccadic displacement paradigms^5^,^30^,^31^,^32^,^33^. In addition to this cancellation property, our data suggest that the same prediction might also facilitate postsaccadic perception under circumstances where nothing changed externally during the saccade. By actively integrating the prediction (based upon prior presaccadic information) with the postsaccadic information, perception can be accurately biased towards presaccadic input. When the world remained stable across a saccade, this could potentially increase sensory sampling efficiency.

The investigation of the time course of transsaccadic integration has thus far mainly focussed on the pre-saccadic acquisition of feature information^6^,^14^,^15^,^17^,^18^. One study showed that presaccadic information is integrated with information that is available upon fixation onset, by measuring participant critical spacing on crowding stimuli^34^. Here, we extend this finding by providing direct empirical evidence that the presaccadically acquired features facilitate postsaccadic perception immediately after saccade offset. This time course is compatible with several reports of early spatiotopic attentional effects briefly after saccade offset^25^,^35^,^36^. Our data are in favour of a visual system that seems to anticipate the consequences of an upcoming saccade in order to readily process postsaccadic visual information using that same prediction^7^,^9^,^37^.

It should be noted that in the current study we did not manipulate or highly restrict the time in which the system could build a spatiotopic prediction before saccade onset. Instead, we allowed subjects to view the peripheral rotating inducer for at least 596 ms (945 ms on average). Interestingly, previous studies suggested that constructing a spatiotopic prediction of visual information might actually take approximately 400 ms^38^,^39^. Here, we did not attempt to investigate the presaccadic build-up of spatiotopic representations. Potentially, saccadic suppression of motion stimuli might reduce the strength of a peripheral inducer when it is only visible shortly before saccade onset, since motion signals are strongly suppressed during saccades^40^. On the other hand, rotational motion is used to induce the High Phi illusion, effectively providing motion energy in all directions. This might minimize suppression, because it has been shown that sensitivity for displacements during saccades is primarily reduced on the axis parallel to the saccade, but not so much in orthogonal directions^41^,^42^. Hence, the paradigm presented here could be a good candidate to investigate the suggested slowly developing spatiotopic representations^39^.

Apart from the time course of integration, it is currently also debated exactly which features are updated spatiotopically across saccades. In Experiment 1 we showed that the accumulated motion information can be stored both in a retinotopic and in a spatiotopic reference frame. The storing of motion information in a retinotopic reference frame is a common finding^19^,^22^. However, spatiotopic updating of accumulated motion information is controversial. On a behavioural level, one study showing spatiotopic motion integration^16^ has been criticized to lack a strict long range control condition that accounted for the presumed spatiotopic effects^21^. Here, we carefully controlled for these potential long range effects, and show that despite the presence of a small long range bias, this cannot fully account for the observed spatiotopic updating of accumulated motion information.

Additionally, spatiotopic updating of motion information has previously been investigated using motion after effects^19^,^22^. The results of those studies suggested that motion cannot be updated spatiotopically, but is strictly represented retinotopically. The retinotopic conditions in Experiment 1 and the control experiment (see Supplementary Information) show that inducer motion energy in the High Phi phenomenon can be similarly accumulated in a retinotopic reference frame across saccades. When the inducer is not rotating retinotopically after the saccade, the effectiveness of the inducer wears off. This might suggest a common mechanism underlying the High Phi phenomenon and motion after effects. However, our findings are in conflict with the conclusion that updating of feature information is restricted to a retinotopic reference frame. Unfortunately, the exact mechanism underlying the High Phi phenomenon remains unknown, so there is no direct explanation why the High Phi illusion can be induced spatiotopically, whereas traditional motion after effects cannot. Hypothetically, High Phi and traditional motion after effects might represent separate phenomena of motion processing. Wexler and colleagues^27^ mention two important differences between the High Phi illusion and traditional motion after effects. First, the amplitude of the perceived jump tends to be equivalent to, or slightly exceeding D_max_, whereas the classical motion after effect tends to have the same amplitude and speed as the inducer^27^. Second, the inducer duration can be very brief for the High Phi illusion, whereas in traditional motion after effects, inducers tend to be effective after longer inducer durations (e.g. ±400 ms for rapid motion after effects)^43^.

How the effects observed here can be explained at the level of neural systems remains a question for future studies. Spatiotopicity of (population) receptive fields in motion sensitive area MT has been suggested^44^,^45^ but debated^46^. More in general, the neural mechanisms of transsaccadic integration are still largely controversial. Classical findings of shifting receptive field around the time of saccades have recently been re-investigated. It was originally interpreted that the visual neurons anticipated the consequences of an upcoming saccade by shifting their receptive fields in the direction of the saccade^1^. However, recently it was shown that the receptive fields actually converge towards the saccade target, instead of linearly shifting in the direction of the saccade^47^. Yet, even more recently, several studies showed at least two different types of shifts in receptive fields: anticipatory vs. memory-based^48^,^49^,^50^. Unfortunately, the link between these neurophysiological findings and the observed behavioural effects in transsaccadic integration still remain unknown (for latest reviews see Higgins and Rayner^51^ or Marino and Mazer^52^). We believe the fast temporal dynamics and the robustness of the effects across subjects show that the current paradigm might provide a valuable tool to further investigate the link between perisaccadic neurophysiology and perception.

To conclude, the current experiments show two main findings. First, accumulated motion information can be updated spatiotopically. Second, presaccadically acquired information influences perception immediately upon saccade landing. The fast, or even instant effect of spatiotopically updated information on postsaccadic perception supports the hypothesis that at least the processing of the saccade target is preceded by a forward model aiming at anticipating the consequences of the eye movement^10^,^11^,^32^,^53^, where the postsaccadic retinal input is predicted based upon the presaccadic retinal input and the characteristics of the upcoming eye movement^8^,^54^.

## Methods

### Subjects

24 subjects (age 18–29, 4 male) with normal or corrected-to-normal vision participated after giving written informed consent. All were naïve to the High Phi illusion and the purpose of the study. 12 subjects participated in Experiment 1, 12 in Experiment 2. This study was approved by the local ethical committee of the Faculty of Social Sciences of Utrecht University. The approved methods were carried out in accordance with the Declaration of Helsinki.

### Setup

Subjects were seated in a darkened room with their heads resting on a chinrest. They were seated 70 cm in front of an LG 24 MB65PM LCD-IPS monitor with a spatial resolution of 1280 × 800 and a refresh rate of 60 Hz. All stimuli were created and presented using Matlab (The MathWorks Inc., Natick MA, 2012) and the Psychophysics Toolbox 3.0^55^,^56^. Eye movements were recorded with an Eyelink 1000 (SR Research Ltd. Ottawa ON; sampling rate of 1000 Hz). The Eyelink was calibrated using the native 9-point calibration routine.

### Stimuli

Subject were presented two annuli with different random textures. One annulus was 7.5° visual angle (VA) to the left of screen centre, the other 7.5° VA to the right. The inner radius of the annuli was 3° VA, the outer 6° VA. In the centre of each annulus was a small fixation point (black, diameter 0.4° VA). The textures of the annuli were random black and white pixels, low pass filtered with circular averaging (bandwidth 1.24 cycles per degree VA). To induce the illusion, the annuli rotated at 20°/sec (Inducer). After the inducer, the texture of the annulus was rapidly replaced by a succession of 4 different, random textures (Transient).

### Procedure Experiment 1

All trials started with a single fixation point combined with the Eyelink 1000 drift check (Fig. 1). A trial started when gaze was closer than 2° to the fixation point and the subject pressed the spacebar. Then, the two annuli appeared, remaining static for 1000 ms (±200 ms, uniformly distributed). In Full match and Long range trials subjects were required to maintain fixation over the entire trial, whereas in Spatiotopic and Retinotopic trials subjects made a saccade. Fixation and saccade trials were presented in separate, interleafed blocks. All trials were flagged invalid and repeated at the end of a block when subjects blinked or when gaze deviated more than 3° VA from fixation during the presentation of the (presaccadic) inducer.

#### Full match

During the presentation of the static annuli, the imminence of the inducer was cued by an auditory beep (261.62 Hz, 50 ms). This beep was not strictly necessary for the task, but was included to keep saccade and fixation trials as similar as possible. Inducer onset was delayed with respect to this cue by 300 ms (±200 ms, uniformly distributed). The inducer rotated for 0, 33.3 1066.7 (60 trials per inducer duration) before transient onset (Fig. 1A). We were primarily interested in the 33.3 and 1066.7 ms conditions, but included the 0 to keep the number of trials in fixation and saccade blocks balanced (see *Data Preprocessing*).

#### Spatiotopic match

After 1000 ms (±200) of static annuli, the peripheral annulus started rotating for 700 ms (Fig. 1B). An auditory cue (440 Hz, 50 ms) instructed subjects to make a saccade to the fixation point at the centre of the peripheral inducer (required saccade amplitude: 15° VA). When gaze was detected within a rectangular area around the peripheral fixation point (width × height: 1° × 4° VA), the inducer kept rotating for an additional 16.7, 33.3 or 50 ms (120 trials) or 1066.7 ms (60 trials) before the transient was presented. We included these 3 possible inducer rotations to obtain a reasonable amount of data points for each participant, when a posteriori computing the postsaccadic inducer duration with respect to saccade offset (see *Data Preprocessing*).

#### Retinotopic match

In retinotopic trials, the presaccadic inducer rotated for 700 ms around fixation, followed by an auditory cue (440 Hz, 50 ms) that instructed the subject to make a saccade to the centre of the peripheral static annulus (Fig. 1C). Postsaccadically, the inducer (now peripheral) kept rotating for an additional 16.7, 33.3 or 50 ms (120 trials) or 1066.7 ms (60 trials) before the transient was presented. Then, the transient was presented around fixation. Thus, after the saccade the inducer and the transient were not matched.

#### Long range

An auditory beep (261.62 Hz, 50 ms) cued the inducer that would be presented 300 (±200) ms later (Fig. 1C). The inducer rotated peripherally for 0, 33.3 or 1066.7 ms and was followed by a transient around fixation.

### Procedure Experiment 2

#### Full match

These trials were similar to the Full match trials in Experiment 1 (Fig. 3A). However, inducer durations were set to 16.7, 33.3 and 50 ms (60 trials per inducer duration). Additionally, the inducer could rotate for 800 ms to verify the effectiveness of the illusion in each subject (60 trials).

#### Spatiotopic match

Procedurally similar to the spatiotopic trials in Experiment 1 with two changes (Fig. 3B). However, we only tested postsaccadic inducer duration of 16.7, 33.3 and 50 ms, randomly drawn from a uniform distribution (180 trials), not 1066.7 ms as in Experiment 1.

#### Saccade cost

These trials were identical to Spatiotopic trials, with the exception that there was no presaccadic inducer (Fig. 3C).

#### Saccade mimic

After the static annuli, a peripheral inducer rotated for 700 ms (±150, uniformly distributed), followed by an auditory beep (Fig. 3D). The peripheral inducer kept rotating for an additional 200 ms (±100 ms), matching the saccadic latencies from Experiment 1. Then, the peripheral annulus stopped rotating and the central annulus rotated for 16.7, 33.3 or 50 ms (60 trials per inducer duration), followed by the transient.

### Screening

In order to verify that subjects could reliably report jump directions, each subject completed a screening task prior to the actual experiment. Here, trials were similar to the Full match trials, with the exception that the transient was substituted by an actual clockwise or counter clockwise rotational jump of 15°. Subjects received feedback on their response: fixation point turned green for correct responses, or red for incorrect responses. All participants performed well above chance on this task (average proportion correct: 0.95, range: 0.75–1.0).

### Data pre-processing

#### Saccade detection

Saccades were detected offline using the Eyelink velocity-based algorithm, with a velocity threshold of 35°/s and an acceleration threshold of 9500°/s^2^. Trials were only included when saccade onset was >100 ms, and the amplitude was >8° VA.

#### Postsaccadic inducer duration

To compute the number of rotational steps of the postsaccadic inducer in frames with respect to saccade offset, we subtracted the time of saccade offset from the time of transient onset. Differences in the interval [16, 32] were considered as a single step, [33, 49] as two steps and [50, 65] as three steps. This calculation was used in order to analyse identical inducer durations in Saccade and Fixation trials. Additionally, in the Saccade Cost condition, we only included trials where the onset of the postsaccadic inducer started within 100 ms upon saccade offset. The median number of trials per subject, per trial type and per inducer duration in frames (and milliseconds) in Experiment 1 and Experiment 2 are summarized in Table 1. Only trials that included a saccade are represented in the table. The fixation conditions contained at least 46 trials per subject per trial type (60 on average).

### Data analysis

Given the imbalance in trial numbers across conditions and subjects we analysed the data using linear logit mixed effects models^28^,^29^. In these models we included Condition and Inducer duration as fixed effects. Subjects were modelled as random offsets. Responses were −1 for backward jumps, and 1 for forward jumps. In Experiment 1, condition and inducer duration were modelled as factors, not numerically. Thus, the expected average response of subject j in condition i is given by


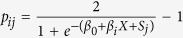


where, *βi* are the coefficients (with one coefficient for each row in the design matrix, and β_0_ is the coefficient in the Full Match condition after 33.3 ms of inducer), *X* is the full factorial design matrix of Trial type (4 levels), Delay (2 levels) and their interaction *S* is the subject-specific coefficient.

In Experiment 1, we did not include 0 ms of inducer because responses are coded with respect to inducer direction. Hence, with no inducer there is no inducer direction to code the responses to.

For the analysis of Experiment 2, a similar model was used. However, here Inducer duration was modelled numerically, from 0 to 2, where 0 represents the baseline of 16.7 ms of inducer.

## Additional Information

**How to cite this article**: Fabius, J. H. *et al*. Spatiotopic updating facilitates perception immediately after saccades. *Sci. Rep.* **6**, 34488; doi: 10.1038/srep34488 (2016).

## Supplementary Material

Supplementary Information

## Figures and Tables

**Table 1:** Median number of trials per inducer duration in the Saccade conditions, with the minimum and maximum number trials across subjects in parentheses.

*Experiment*	*Condition*	*Inducer duration in frames (ms*)			
		1 (16.7)	2 (33.3)	3 (50)	64 (1066.7)
1	Spatiotopic	—	30.5 (16–38)	—	27 (12–43)
	Retinotopic	—	33 (26–42)	—	27.5 (15–42)
2	Spatiotopic	45.5 (15–67)	49 (34–64)	35.5 (13–56)	—
	Saccade cost	26.5 (11–55)	44.5 (30–57)	39 (19–60)	—
