# Supplementary material for: Spatiotopic updating facilitates perception immediately after saccades
Source: Sci Rep. 2016 Sep 30;6:34488. doi: 10.1038/srep34488 (PMC5043283; doi:10.1038/srep34488)
Supplement: Supplementary Information [file srep34488-s1.pdf]

Spatiotopic updating facilitates perception immediately after saccades
[Supplementary Information]

Jasper H. Fabius[1*+], Alessio Fracasso[1,2,3+] & Stefan Van der Stigchel[1]

Control experiment – Purely Retinotopic

Methods

All included subjects scored at least 75% correct on the screening (as described in the paper). Two additional subjects were tested, but failed to reach this inclusion threshold. They therefore did not perform the experimental conditions. Similar to Experiment 1 and 2, all trials started with a single fixation point combined with the Eyelink 1000 drift check and required the subjects to start the trial by pressing the spacebar. Then, the two annuli appeared, remaining static for 800-1000. After that each trial proceeded differently. Trials in the Full Match and Spatiotopic condition were similar to the same conditions in Experiment 1 and 2.

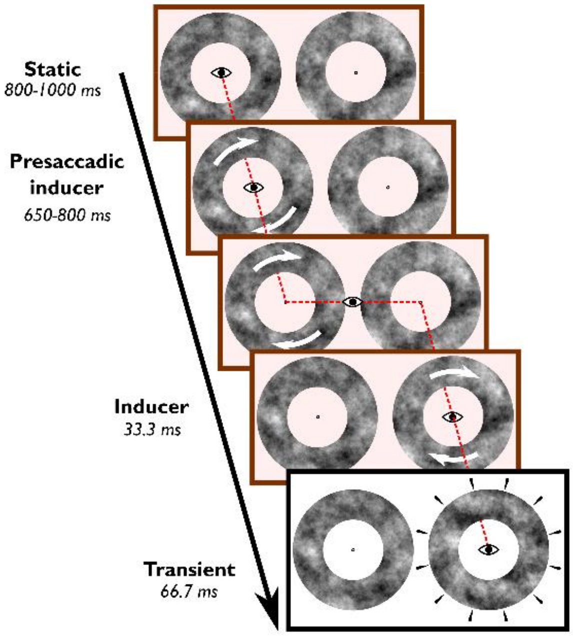

Figure S1. Purely Retinotopic trial. The position of the presaccadic inducer was initially around fixation. After an auditory cue, a saccade was executed and the inducer motion was shifted along with the saccade. After the saccade, the inducer would rotate for another 33.3 ms. Then, the transient was always presented around the last fixation point.

1. **Full Match** – In these trials, the annuli remained static for another 650-800 ms. Then the auditory beep was played, and 200-300 ms later the inducer was presented. The inducer rotated for 33.3 or 800 ms before transient onset.
2. **Spatiotopic** – The peripheral annulus started rotating for 650-800 ms. Then the beep was played, and subjects made a saccade towards the rotating annulus. After gaze was detected within a rectangular ROI (1x4° VA) the inducer kept rotating for another 33.3 or 50 ms. A posteriori we determined the actual inducer duration with respect to saccade offset.
3. **Purely retinotopic** – The annulus around fixation rotated for 650-800 ms. Then the beep was played, and subjects made a saccade towards the rotating annulus. After gaze was detected within a rectangular ROI (1x4° VA) the other annulus, (now around fixation) rotated for 33.3 or 50 ms. The annulus that rotated initially stopped rotating when the other started (Figure S1).

Results

Data preprocessing – After setting transient onset with respect to inducer onset we had on average 20 trials per subject in the spatiotopic condition (range: 17-26) and 20 trials per subject in the retinotopic condition (range: 13-29).

Perceived jump direction – We analyzed the effects of condition in the trials with short inducers (33.3 ms) on the perceived jump direction in a linear mixed effects analysis (Figure S1). The reported effects are reported in reference to the Full Match condition. Like in the other experiments, 33.3 ms of inducer was sufficient to produce a bias in perceived jump direction (β = -0.92, z = 5.28, p < 0.001), and this bias was stronger when a spatiotopic preview of the inducer was provided (β = -1.15, z = 4.33, p < 0.001). Moreover, like in Experiment 1, the observed bias was also stronger when a retinotopic preview was provided (β = -1.08, z = 4.11, p < 0.001). There was no significant difference between the Spatiotopic and Retinotopic conditions (β = 0.08, z = 0.22, p = 0.83). To test our hypothesis more directly, we also compared both 'saccade'-conditions to the Full Match trials where the inducer rotated for 48 frames. There was a very strong bias in these Full Match trials (β = -3.32, z = 8.89, p < 0.001), that was stronger than the bias in both the Spatiotopic (β = 1.03, z = 3.10, p = 0.002) and the Retinotopic trials (β = 1.13, z = 3.42, p < 0.001). The difference between the Retinotopic trials and the long Full Match trials might be explained by a potential cost of the intervening saccade.

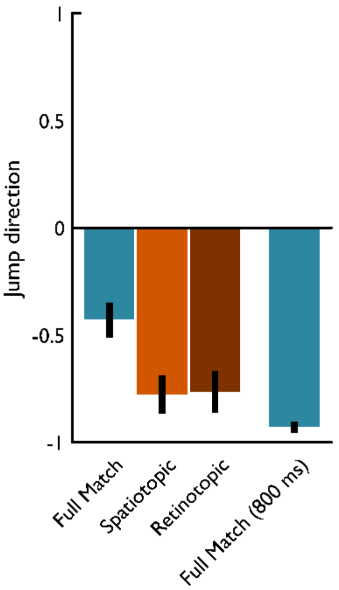

Figure S2. Perceived jump direction in Experiment 3. Error bars represent bootstrapped 95%-confidence intervals of the model estimates. The three left bars, are the average response after 33.3 ms of (post-saccadic) inducer.

Control analysis – Fixation position

As can be seen in Figure 5a in the main text, there was more variance in the average fixation positions during transient presentation between the different conditions. We used Levene's test to test the differences in horizontal variance of the average fixation positions. First, we compared the conditions with saccades versus conditions without in Experiment (Full Match and Long Range versus Spatiotopic and Retinotopic). There was more variance in the conditions with saccades ($F(1) = 58.03$, $p < 0.001$). However, there was no difference in the variance of the Full Match and the Long Range condition ($F(1) = 0.01$, $p = 0.91$), nor between the Spatiotopic and the Retinotopic condition ($F(1) = 0.41$, $p = 0.52$). We followed the same procedure for Experiment 2 (Full Match and Saccade Mimic versus Spatiotopic and Saccade Cost). Again, there was a difference between the conditions with and without saccades ($F(1) = 322.96$, $p < 0.001$), but not between the different types of fixation ($F(1) = 0.56$, $p = 0.46$) or saccade conditions ($F(1) = 2.73$, $p = 0.10$).

Given the difference in fixation position during the presentation of the transient between the conditions with and without saccade we analyzed to more measures of fixation position.

1. *Fixation variance*. This is a measure of the stability of fixation. We defined fixation variance as the average distance of raw x-y gaze coordinates from average gaze position during transient presentation (per trial): $\sqrt{(x - \bar{x})^2 + (y - \bar{y})^2}$.
2. *Fixation error*. This is a measure of the retinal mismatch of the annuli around fixation during the presentation of the (pre-saccadic) inducer and during the transient. We defined fixation error as the distance between the fixation position during the presentation of the (presaccadic) inducer and the transient. These are positions are taken with respect to the fixation dot: $\sqrt{(\bar{x}_{transient} - \bar{x}_{inducer})^2 + (\bar{y}_{transient} - \bar{y}_{inducer})^2}$.

Experiment 1

Fixation variance (Figure S3, top left) – We constructed linear mixed effects models for the variance in fixation during transient presentation, with condition as a fixed effect and subject as a random effect. The Full Match condition was taken as the reference level. This analysis showed a difference in average spread of coordinates between trials with saccades (Spatiotopic and Retinotopic conditions) and trials without (Full Match and Long Range conditions). The average spread during presentation of the transient was 1.17° VA in the Full Match trials ($t = 23.26$) and not significantly different from Long Range trials ($\beta = 0.02°$ VA, $t = 0.69$). In Spatiotopic trials this spread was 0.18° VA larger ($t = 4.87$), in Retinotopic trials 0.16° VA ($t = 4.49$).

Fixation error (Figure S3, bottom left) – Another linear mixed effects model was constructed to analyze fixation error (as defined above), with condition as a fixed effect and subject as a random effect. In Full Match trials there was only a small difference between average fixation position during the inducer and during the transient (β = 0.12° VA, t = 4.63), not significantly different from the fixation error in Long Range trials (β < -0.01° VA, t = 0.59). The fixation error was 0.61° VA larger in Spatiotopic trials (t = 46.41) and 0.67° VA larger in Retinotopic trials (t = 52.08).

Fixation variance and error as random effects – We added random slopes of the trial by trial fixation variance (as defined above) and fixation error (as defined above) within each subject as two random effects to our original logit linear mixed effects model. We compared the models with and without (the original model) with a log likelihood test. The additional random effects did not improve the fit of the model ($\chi^2(5) = 3.22$, p = 0.67).

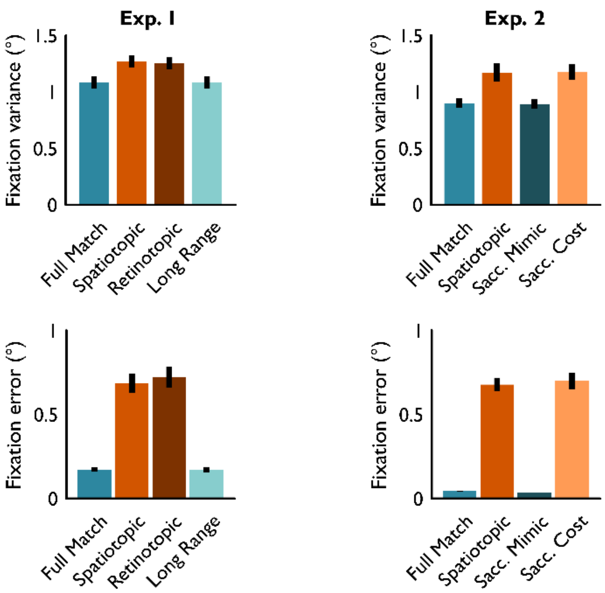

Figure S3. Fixation parameters in Experiment 1 and 2
Top panels: average fixation variance across subjects (± s.e.m.) for the different conditions in Experiment 1 (left) and Experiment 2 (right). This is a measure of the stability of fixation.
Bottom panels: average fixation error across subjects (± s.e.m.) for the different conditions in Experiment 1 (left) and Experiment 2 (right). This is measure of the retinal mismatch of the annuli around fixation during the presentation of the (pre-saccadic) inducer and during the transient.

Experiment 2

Fixation variance (Figure S3, top right) – We followed the same analysis procedure for Experiment 2 as for Experiment 1. This analysis showed a difference in average spread of coordinates between trials with saccades (Spatiotopic and Retinotopic conditions) and trials without (Full Match and Long Range conditions). The average spread during presentation of the transient was 1.00° VA in the Full Match trials (t = 16.12) and not significantly different from Saccade Mimic trials (β = -0.02° VA, t = 0.91). In Spatiotopic trials this spread was 0.31° VA larger (t = 11.18), similar to the Saccade Cost trials (β = 0.30° VA, t = 11.01).

Fixation error (Figure S3, bottom right) – In Full Match trials there was only a small difference between average fixation position during the inducer and during the transient (β = 0.04° VA, t = 2.34), not significantly different from the fixation error in Long Range trials (β < -0.01° VA, t = 0.10). The fixation error was 0.70° VA larger in Spatiotopic trials (t = 80.44) and 0.69° VA larger in Retinotopic trials (t = 77.27).

Fixation variance and error as random effects – Again, we added the trial by trial fixation variance and fixation error to our original logit linear mixed effects model of Experiment 2. We compared the models with and without (the original model) with a log likelihood test. In contrast to Experiment 1, the additional random effects did improve the fit of the model ($\chi^2(5) = 16.28$, $p = 0.007$). However, inferences based on the estimated parameters stay the same as without the random effects (Table S1).

Table S1.
Comparison of fixed effects in the original model (as used in the manuscript) and with the addition of two random effects: fixation variance and fixation error. Although the model with the two additional random effects fits the data better, the inferences on the fixed effects are similar for both models.

	**Model 1: response ~ condition * inducer duration + (1 | subject)**			**Model 2: response ~ condition * inducer duration + (1 + fixation variance + fixation error | subject)**		
**Fixed effect**	**β value**	**z value**	**p value**	**β value**	**z value**	**p value**
**Intercept (Full Match)**	0.02	0.15	0.882	0.02	0.16	0.876
**Spatiotopic**	-1.33	-10.78	< 0.001	-1.30	-9.57	< 0.001
**Saccade Mimic**	-0.37	-3.74	< 0.001	-0.37	-3.75	<0.001
**Saccade Cost**	0.02	0.15	0.878	0.05	0.43	0.665
**Inducer duration**	-0.65	-11.18	< 0.001	-0.66	-11.21	< 0.001
**Inducer duration:Spatiotopic**	0.14	1.24	0.215	0.13	1.20	0.230
**Inducer duration:Saccade Mimic**	0.10	1.17	0.242	0.10	1.18	0.238
**Inducer duration:Saccade Cost**	0.27	3.01	0.003	0.27	2.91	0.004
